# Influence of Heat Treatment on Microstructure, Mechanical Property, and Corrosion Behavior of Cold-Sprayed Zn Coating on Mg Alloy Substrate

**DOI:** 10.3390/ma15196721

**Published:** 2022-09-27

**Authors:** Zhenpeng Zhou, Xiao Chen, Xiaozhen Hu, Sheng Li, Menglong Lv, Yiting Xie, Hailong Yao, Hongtao Wang, Xiaobo Bai

**Affiliations:** 1 Jiangxi Province Engineering Research Center of Materials Surface Enhancing & Remanufacturing, School of Materials Science and Engineering, Jiujiang University, Jiujiang 332005, China; 2 Xinyu Key Laboratory of Materials Technology and Application for Intelligent Manufacturing, School of Mechanical and Electrical Engineering, Xinyu University, Xinyu 338004, China; 3 School of Architecture Engineering and Planning, Jiujiang University, Jiujiang 332005, China

**Keywords:** cold spray, Zn coating, heat treatment, microstructure, mechanical property, corrosion behavior, biodegradable Mg alloy

## Abstract

The influence of post-process heat treatment on cold-sprayed Zn coatings on the Mg alloy substrate was investigated at different temperatures (150, 250, and 350 °C) and times (2, 8, and 16 h). Phase, microstructure, microhardness, and tensile strength of Zn coatings were analyzed before and after heat treatment. Corrosion properties of Zn coatings after heat treatment were investigated in simulated body fluid by using potentiodynamic polarization and immersion testing. Results show that although the heat treatment presented little effect on phase compositions of Zn coatings, the full width at half maxima of the Zn phase decreased with the heat temperature and time. Zn coatings presented comparable microstructures before and after heat treatment in addition to the inter-diffusion layers, and the inter-diffusion layer was dependent on the heat temperature and time. Both the thickness and the microhardness of inter-diffusion layers were increased with the heat temperature and time, with the largest thickness of 704.1 ± 32.4 μm and the largest microhardness of 323.7 ± 104.1 HV_0.025_ at 350 °C for 2 h. The microhardness of Zn coating was significantly decreased from 70.8 ± 5.6 HV_0.025_ to 43.9 ± 12.5 HV_0.025,_ with the heat temperature from the ambient temperature to 350 °C, and was slightly decreased with the heat time at 250 °C. Although the tensile strength of Zn coating was slightly increased by heat treatment, with the highest value of 40.9 ± 3.9 MPa at 150 °C for 2 h, excessive heat temperature and time were detrimental to the tensile strength, with the lowest value of 6.6 ± 1.6 MPa at 350 °C for 2 h. The heat temperature and heat time presented limited effects on the corrosion current and corrosion ratio of the Zn coatings, and Zn coatings before and after heat treatment effectively hindered the simulated body fluid from penetrating into the substrate. The corrosion behavior of Zn coatings was discussed in terms of corrosion products and microstructures after immersion.

## 1. Introduction

Magnesium alloys present excellent biodegradability and suitable mechanical properties to natural bones, which have been considered novel potential implants [1]. The current use of Mg alloys is restricted mainly to the cardiovascular stent scaffold, bone repair, and fixation materials [1,2]. However, Mg alloys have rapid corrosion rates in the physiological environment and produce hydrogen gas [3,4]. These shortcomings result in unpredictable failures of Mg-based implants and limit the actual application for load-bearing implant materials. The controlled degradation of Mg alloys makes them suitable for degradable biomaterial. Bulk alloying, microstructure modification, and surface coatings are effective and widely used strategies to improve the anti-corrosion properties of Mg alloys [3,4]. Surface coatings can hinder the corrosion medium from penetrating into the Mg alloy’s surface and decrease their corrosion rates [1,5]. Considering the advantages of biodegradability for Mg alloys, the surface coatings should also be biodegradable and have lower corrosion rates than the Mg alloy substrates.

Mg, Zn, and Fe are considered biodegradable metals, and Zn and Fe present higher corrosion resistance than Mg [6,7]. Zinc is an essential element in the human body, which plays a positive role in the mineralization of bones and cellular protein synthesis [7,8]. When compared with ceramic and organic coatings, Zn coating presents comparable mechanical properties with Mg alloys. This can avoid the cracking and peeling-off of Zn coatings on the surface of Mg alloy. Therefore, the zinc coating is the potential and biodegradable strategy to improve the anti-corrosion of Mg alloys for implants.

Cold spray is a novel and environmentally-friendly technology, which can produce metal, ceramic, cermets, and organic coatings on the surface of different materials [9,10]. Unlike other thermal spraying techniques, solid-state particles at a low temperature are accelerated to a high velocity to impact the substrate and form a dense, non-oxidized, and adherent coating. These features solve the common drawbacks of conventional thermal spraying techniques for oxygen-sensitive or thermo-sensitive coating and substrates [9,10]. Many studies have explored the microstructures and properties of Zn coatings deposited by cold spray. Naveen et al. report that cold-sprayed Zn coatings improved the corrosion resistance of the mild steel substrate [11]. Maledi et al. explore the residual stresses of cold-sprayed Zn coating on a mild steel substrate [12]. Li et al. address that the impact-induced melting significantly enhances the coating/substrate interface bonding and inter-particles bonding in the cold-sprayed Zn coating [13]. The authors also investigate the corrosion resistance and bioactivity of Zn coating and Zn/HA composite coatings on the Mg alloy substrate [14,15].

Cold-sprayed metal coatings are formed by plastic deformations of metal particles due to the high-velocity impact. The inter-particle bonding interface and the coating/substrate interface are weak and mechanically interlocked, which results in overall poor ductility with brittle fractures commonly occurring [16,17]. To attain higher bonding strength and promote the overall coating performance, post-process heat treatment is usually conducted on the cold-sprayed coating. Mechanical properties and corrosion resistance of cold-sprayed Zn coating on mild steel substrate are improved by post-process heat treatment due to the improvement of both the inner particle interface and coating/substrate interface [11]. For Mg alloy substrates, heat treatment can result in element diffusions to form intermetallic compounds within Al/Mg [18,19], Cu/Mg [20], and Zn/Mg couples [21]. In an Mg/Zn couple, intermetallic compounds are also observed in the Mg-Zn inter-diffusion layer after heat treatment [21,22,23,24,25]. The square of thickness for the inter-diffusion layer increases linearly with heat time [24] and the pressure can promote the formation of intermetallic layers [21]. Intermetallic compounds always present higher microhardness than the substrates [21,22,23,24,25], but the inter-diffusion layer with intermetallic compounds presented positive and negative effects on the corrosion properties [21]. However, the intermetallic layer is never reported in the cold-sprayed Zn coatings on Mg alloy substrate, although the heat treatment of cold-sprayed Zn coating is reported without any diffusion layer at the Zn coating/steel interface [11]. Therefore, it is necessary to investigate the effect of heat treatment on the cold-sprayed Zn coating on Mg alloy substrate.

This work aimed to investigate the influence of heat temperature and time on microstructure, mechanical, and anti-corrosion properties of cold-sprayed Zn coatings on Mg alloy substrates. The formation of the intermetallic layer at the Zn coating/Mg alloy substrate interface and corrosion behaviors of Zn coatings after heat treatment were discussed.

## 2. Experimental Procedure

### 2.1. Coatings Preparation and Heat Treatment

Spherical zinc powder (size 15–53 μm, 98.8 wt.% Zn, 1.2 wt.% O on the powder surface, Beijing Youxinlian Nonferrous Metals Co. Ltd., Beijing, China) was used as the original material of Zn coatings (as shown in reference [14]). Spherical 1Cr18 stainless steel (S30210, GB/T 20878) particles (size range from 350 to 500 μm) were used as shots. Some 50 vol.% zinc powders and 50 vol.% shots were mixed as a starting powder in a mechanical mixer for 12 h. Cast AZ31B Mg alloy plates (94.21 wt.% Mg, 2.48 wt.% Al, 3.31 wt.% Zn, dimension 10 × 10 × 4 mm) were utilized as substrates. Before the deposition of coatings, the substrates were sand-blasted with Al_2_O_3_ grit (200 μm) for 5 min and then cleaned in ethanol. The Zn coatings were deposited on AZ31B substrates by a cold-spraying system assembled by Xi’an Jiaotong University. Both the primary and carrier gases were made of nitrogen. The primary gas pressure and temperature were 2.0 ± 0.1 MPa and 260 ± 20 °C, respectively. The spray distance was 20 mm, and the transverse speed of the torch was 50 mm/s. After coating deposition, zinc coatings were put into a silica tube. Then the tube was sealed in a vacuum and heated up to 150 °C for 2 h, 250 °C for 2, 8, 16 h, and 350 °C for 2 h.

### 2.2. Phase and Microstructure Characterization

The phase structures of different Zn coatings were identified by X-ray diffraction (XRD, D8 Advance, Bruker, Ettlingen, Germany) at Cu_Kα_ radiation of 1.5418 Å, an operating voltage of 35 kV, an operating current of 35 mA, a 2θ range of 20–90°, and a scan rate of 0.1°/s. Cross-sectional morphologies of different Zn coatings were investigated by scanning electron microscopy (SEM, VEGA II, Tescan, Brno, Czech Republic). The chemical elements were examined by an energy-dispersive spectrometry (EDS) module integrated within SEM.

### 2.3. Microhardness and Bond Adhesion Tensile Strength Test

Microhardness of Zn coatings before and after heating was obtained by Vickers microhardness tester (HVS-1000, Shanghai Precision Instruments Co., Ltd., Shanghai, China) at the load of 25 gf and the dwell time of 20 s. Tensile strengths of Zn coatings before and after heating were measured using a standard tensile test method (ASTM-C633) designed for thermal sprayed coatings. The end surface of the cylindrical sample with Zn coatings was boned on grit-blasted facings of the loading fixtures, being the same size and shape as the sample, using a special adhesive glue (E-7, Adtest, Shanghai Huayi Resins Co. Ltd., Shanghai, China) with a tensile strength of about 70 MPa. The assembly was held perpendicularly and placed in an oven at 100 °C for 2 h. After the bonding glue was cured and hardened, the assembly was loaded into the machine (ZWICK, Z050 model, Ulm, Germany) at a head speed of 1 mm/min. The bond strength of coatings was evaluated by the average value of at least three sample test results. After tensile testing, the surface and cross-sectional microstructures of fractured Zn coatings were examined by SEM.

### 2.4. Corrosion Testing

The potentiodynamic polarization tests were carried out in simulated body fluid (SBF) at the temperature of 37 ± 1 °C (Modified-SBF, Qingdao Jisskang Biotechnology Co. Ltd, Qingdao, China). The SBF contained 142.0 mM Na^+^, 5.0 mM K^+^, 1.5 mM Mg^2+^, 2.5 mM Ca^2+^, 103.0 mM Cl^−^, 10.0 m HCO_3_^−^, 1.0 mM HPO_4_^2−^, and 0.5 mM SO_4_^2−^. Before testing, the Zn coatings before and after heating were immersed in SBF for 1, 7, and 14 days. Polarization curves were then carried out on an electrochemical workstation (IM6, Zahner, Kronach, Germany) from voltages of −2 V to 1 V (vs. SCE) at a scan rate of 1 mV/s. At least three tests were carried out for each sample. After immersion for 14 days, the surface and cross-sectional microstructures, and phase constitutions of Zn coatings were examined by SEM and XRD, respectively.

## 3. Results and Discussion

### 3.1. Phase Composition

**Figure 1:**
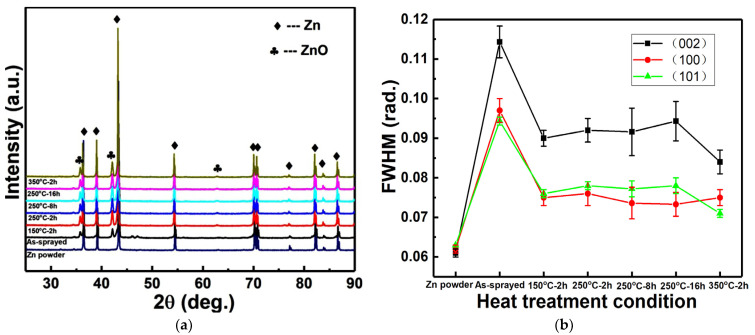
XRD patterns and FWHM of Zn coatings after different heating conditions. (**a**) XRD patterns; (**b**) FWHM of Zn phase.

Figure 1a shows XRD patterns of Zn powder and Zn coatings before and after different heat conditions. Compared with the Zn powder, Zn coatings were mainly composed of Zn in addition to a small amount of ZnO. It indicates the oxidation of Zn particles during coating depositions, which may be attributed to a low melting point of Zn. It is also reported that the impact can induce the melting of Zn particles [13], and melting Zn particles can react with the oxygen in the atmosphere. After heat treatment, there was no significant effect of both the heat temperature and time on phase compositions of Zn coatings. However, there was an obvious difference in the diffraction peaks of the Zn phase. Figure 1b shows the full width at half maxima (FWHM) of (002), (100), and (101) diffraction peaks of the Zn phase. All the Zn coatings show higher FWHM than the Zn powder, and Zn coatings after heat treatment show lower FWHM than the as-sprayed Zn coating. FWHM is typically relative to the grain refinement, residual stress, and plastic deformation within the cold-sprayed metal coatings [16,26]. Peak broadening, as well as the increase in FWHM, can be attributed to an accumulation of stacking faults and structural disorders, grain refinement as well as increased stress [12]. This decrease in the FWHM can be attributed to the release of internal stress within the microstructure, recrystallization, and grain growth after heating. Therefore, although the heat treatment presented little effect on phase compositions of Zn coatings, the full width at half maxima of the Zn phase was decreased with the heat temperature and time.

### 3.2. Cross-Sectional Microstructures after Heat Treatment

**Figure 2:**
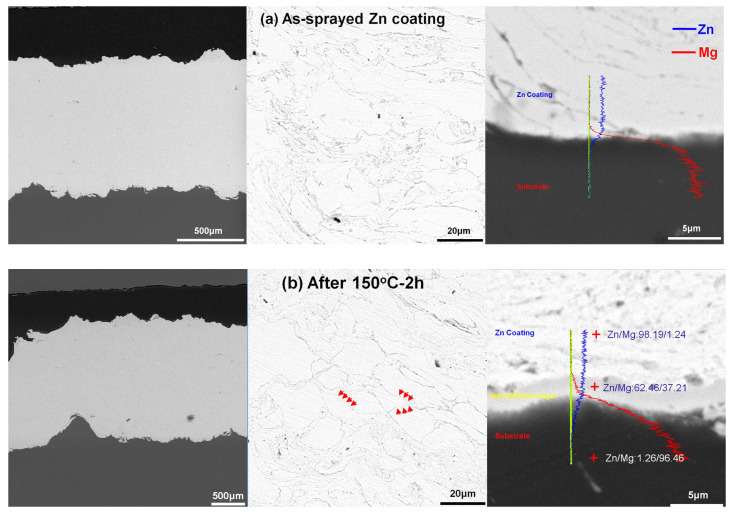

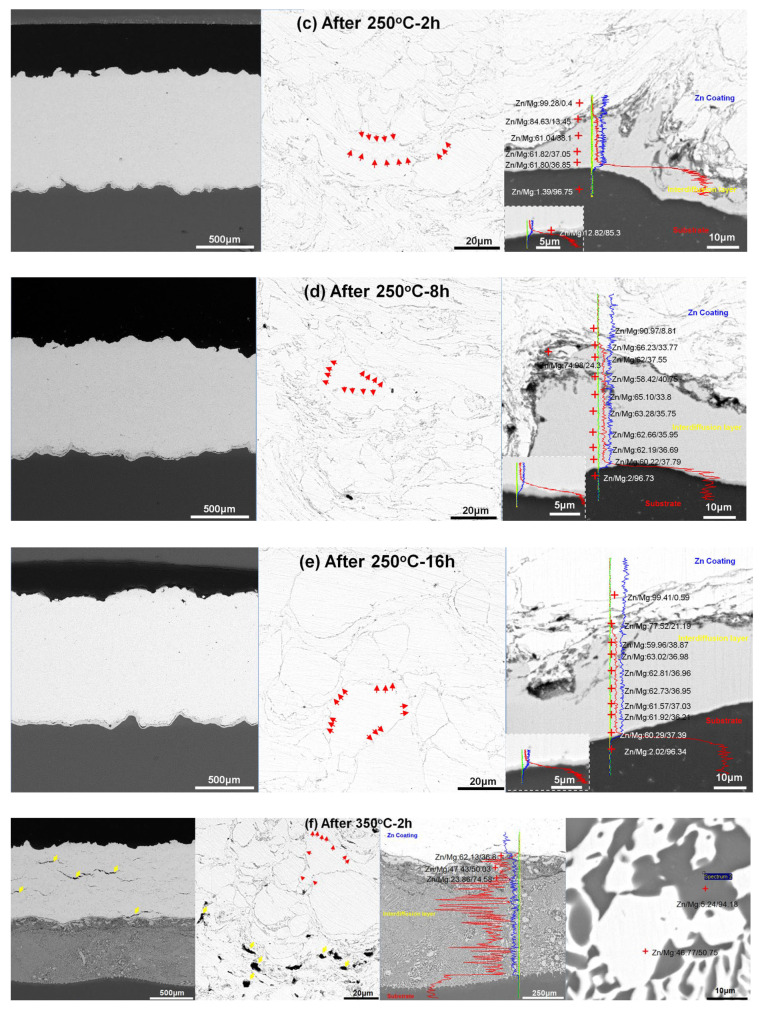
Cross-sectional microstructures of Zn coatings after different heating conditions. Red line: Mg K_α__1-2;_ Blue line: Zn K_α__1_; Green line: Al K_α__1_.; Yellow line: EDS scan trace.

Figure 2 shows cross-sectional microstructures of Zn coatings before and after different heat conditions. It can be found that the as-sprayed Zn coating presented a well-bonded coating/substrate interface and a very low porosity in Figure 2a. Compared with the spherical Zn powder, irregular Zn particles with severe plastic deformations were attributed to the high-velocity impact and hammering effect induced by the shot peens [27,28,29]. After different heat conditions in Figure 2b–f, Zn coatings were bonded to the Mg alloy substrate without obvious microcracks and pores, except for the Zn coating heated at 350 °C for 2 h in Figure 2f. In Figure 2, bright Zn particles with gray boundaries were observed in all the Zn coatings. From the high-magnified back-scattered electronic images, the gray boundary was mainly composed of O and Zn atoms, and the light region was only composed of Zn atoms. According to the XRD patterns, it indicates that the gray boundary was composed of the ZnO phase and the light region was Zn. It can be found that after heat treatment, some gray boundaries between Zn particles disappeared with the diffusion of O into Zn particles, as shown by red arrows in Figure 2. This may indicate that particle bonding occurred and a metallurgical bonding between the Zn particles was formed as reported for cold-sprayed pure Cu and Cu-based alloy coatings [30]. In addition, some large pores and microcracks were observed in the Zn coating heated at 350 °C for 2 h, as tabbed as yellow arrows in Figure 2f. The formation of microcracks could be due to the mismatch of mechanical properties between the Zn coating, inter-diffusion layer, and Mg alloy substrate. In addition, the internal stress induced by the grain growth of Mg alloy substrate could also result in cracks. These large pores could result from the coalescence of the incomplete interfaces in the as-sprayed coatings through atom diffusions during the heat treatment with relatively high temperatures. Therefore, the heat treatment may improve the inter-particle bonding rather than the coating/substrate interface bonding.

**Figure 3:**
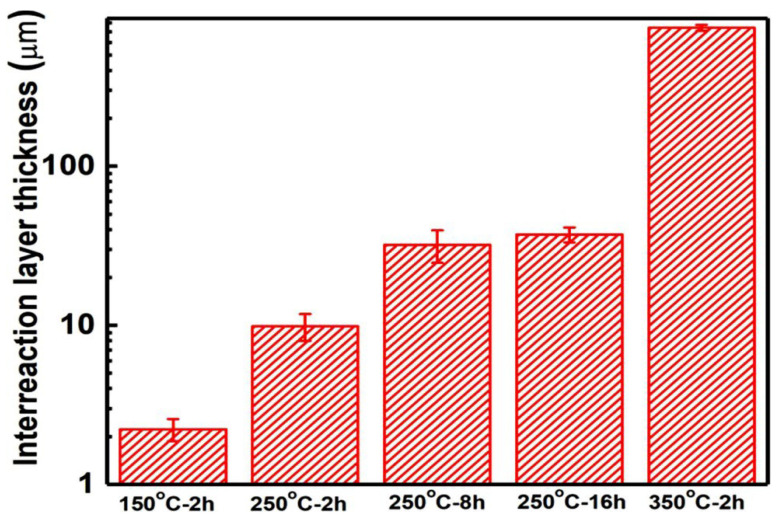
Thickness of inter-diffusion layers in Zn coatings after different heating conditions.

There were obvious inter-diffusion layers between Zn coatings and the Mg alloy substrate. The formation of a diffusion layer is reported for a Mg alloy brazed joint using Zn-based filler metal [21,22,31,32,33]. The thickness of the inter-diffusion layer was increased with the heat temperature and was slightly increased with the heat time as shown in Figure 3. In addition to the different thicknesses of inter-diffusion layers, there were significant differences between the diffusion layer/Zn coating interface and the diffusion layer/Mg alloy substrate interface. The different diffusion coefficient between the Zn atom and the Mg atom results in the formation of pores and microcracks at the side of Zn coatings [23,24]. At the diffusion layer/Zn coating interfaces, some pin pores and microcracks were observed at a heat temperature higher than 150 °C. While the diffusion layer/substrate interfaces show uniform and dense microstructures without any pores and microcracks, EDS scan lines show distributions of the Zn and Mg atoms at the Zn coating/diffusion layer/Mg alloy substrate interfaces, as shown in Figure 2. It can be found that the content of the Zn atom gradually decreased from the Zn coating to the Mg alloy substrate and the content of the Mg atom showed the opposite. Meanwhile, the Zn shows a higher content than Mg in the diffusion layer. According to the Mg-Zn phase diagram [24], several intermetallic phases could be produced at the diffusion layer i.e., Mg_21_Zn_25_, Mg_2_Zn_3_, MgZn_2_, and Mg_2_Zn_11_. The Mg/Zn atom ratios at different regions along the inter-diffusion layers were presented in Figure 2. It can be deduced that the diffusion layer was composed of three different regions for Zn coating after heating at 150 °C and 250 °C, i.e., the region close to Zn coating was the Mg_2_Zn_11_ phase, the middle region was the MgZn_2_ and Mg_2_Zn_3_ phase, and a very thin transition layer was closed to Mg substrate. According to the Mg-Zn phase diagram [24], the eutectic reaction between Zn and Mg can occur at 340 °C with the Zn content from 10 wt.% to 80 wt.%. For the heating condition of 350 °C for 2h, the eutectic reaction could occur at the Zn coating/Mg alloy substrate interface. EDS result shows the compositions of different regions in the reaction layer. It can be deduced that the eutectic layer was mainly composed of α-Mg and Mg-Zn intermetallic phase, which is reported in the Mg alloy brazed joint using a Zn-based filler metal at a high temperature [31]. Therefore, except for 350 °C for 2 h, it can be considered that Zn coatings presented comparable microstructures before and after heat treatment in addition to the inter-diffusion layers, and the inter-diffusion layer was dependent on the heat temperature and time.

### 3.3. Microhardness after Heat Treatment

**Figure 4:**
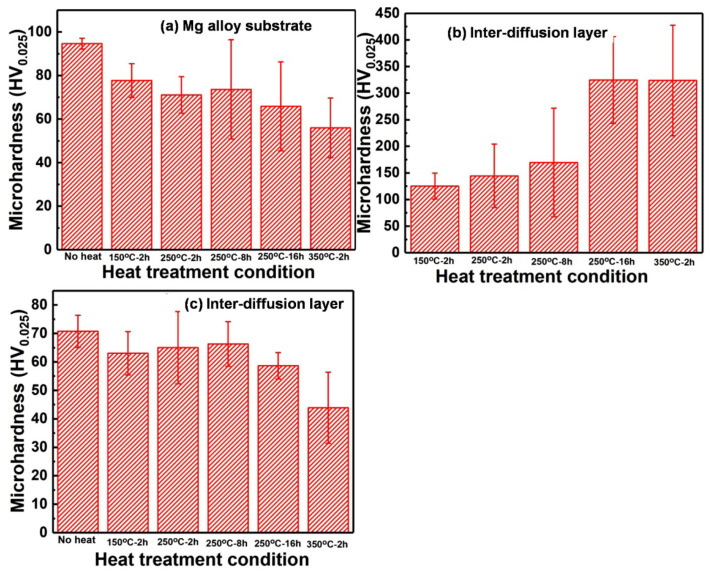
Microhardness of Zn coatings after different heating conditions.

Figure 4 shows the microhardness of Mg alloy substrate, Zn coatings, and inter-diffusion layers after different heating conditions. It can be seen that both the Mg alloy substrate and Zn coatings show a decrease in microhardness with heat temperature and heat time. The decrease in microhardness of the Mg alloy substrate can be mainly due to the dissolution of the hard β phase, which acts as a reinforcement phase in the magnesium alloy matrix [19]. The decrease in microhardness of Zn coatings can be attributed to the release of internal stress and dislocation density during the post-treatment. By extending the heat time, the decrease in microhardness of Zn coatings may be mainly attributed to the recrystallization and grain growth [18]. Therefore, both the Zn coatings and Mg alloy substrate presented softening after heat treatment. However, the inter-diffusion layer shows an opposite evolution of microhardness and higher microhardness than the Mg alloy substrate and the Zn coatings. Mg-Zn intermetallic compounds (Mg_21_Zn_25_, Mg_2_Zn_3_, MgZn_2_, and Mg_2_Zn_11_) exhibit significantly higher microhardness compared with the pure Zn and Mg [24]. It is also reported that the microhardness of an α-Mg+Mg/Zn eutectoid structure is much higher than that of an α-Mg solid solution [31]. The increase in the microhardness of diffusion layers was due to the formation of intermetallic compounds. The low microhardness of diffusion layers at the heating conditions of 150 °C-2h, 250 °C-2h, and 250 °C-8h could be attributed to the thin diffusion layers [32]. It can be concluded that inter-diffusion layers with intermetallic compounds formed after heat treatment lead to high microhardness in the Zn coating/Mg alloy substrate interface.

### 3.4. Tensile Strength after Heat Treatment

**Figure 5:**
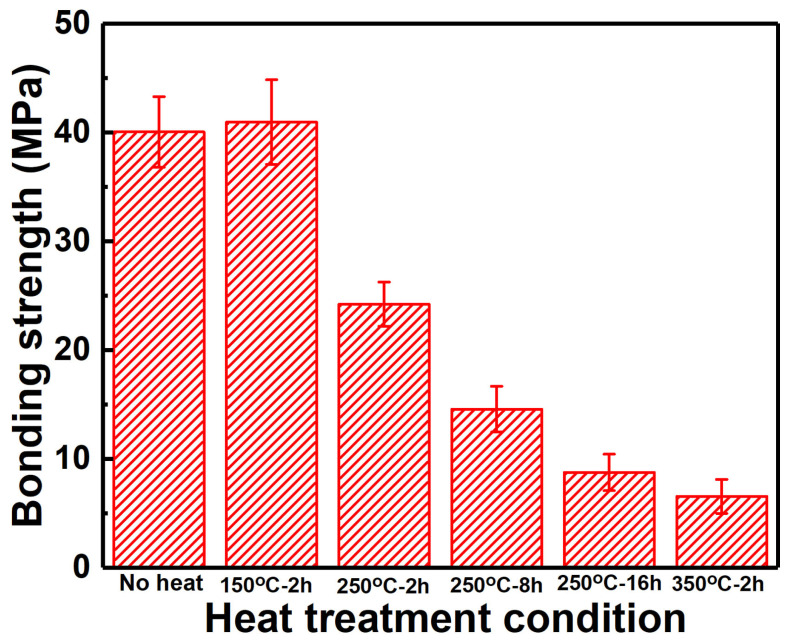
Bonding strength of Zn coatings after different heating conditions.

Figure 5 shows the tensile strength of Zn coatings before and after heating. It can be found that in addition to the slight increase in tensile strength for the Zn coating heated at 150 °C for 2 h, other Zn coatings after heat treatment presented decreases in the tensile strength. The Zn coating heated at 350 °C for 2 h presented the lowest tensile strength of 6.6 ± 1.6 MPa. This increased tensile strength of Zn coating after heating at 150 °C for 2 h was consistent with the reported result [13], but the increase in heat temperature and time was detrimental to the tensile strength. It is reported that the mechanical interlocking between the metal particles determines the mechanical properties of the cold-sprayed metal coatings [34,35]. After heat treatment, the metallurgical bonding between the deposited particles can be formed and the tensile strength is commonly increased [18,19,33,36]. Therefore, although the tensile strength was improved by the heat treatment, excessive heat temperature and time are detrimental to the tensile strength of cold-sprayed Zn coatings.

**Figure 6:**
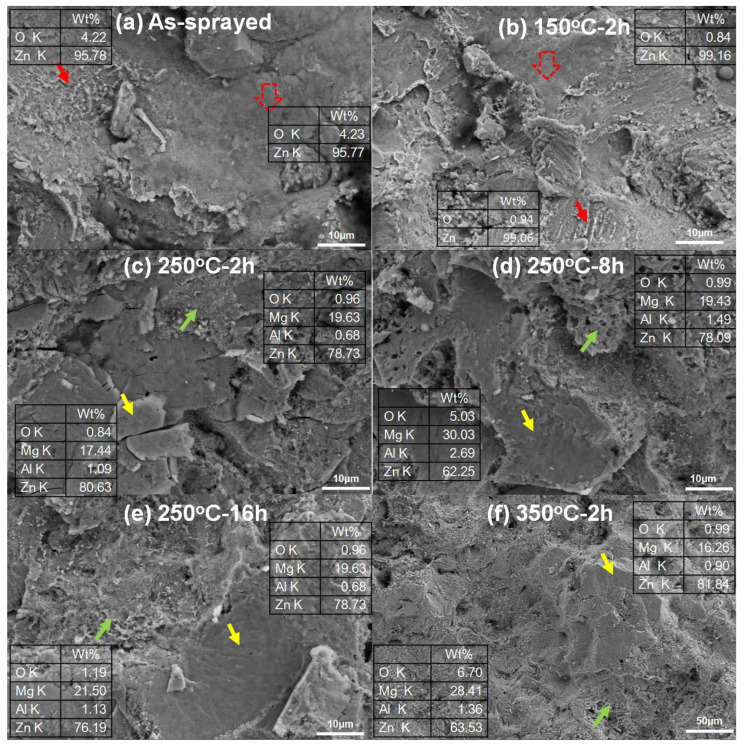
Morphologies of fractured surfaces of Zn coatings after different heating conditions.

**Figure 7:**
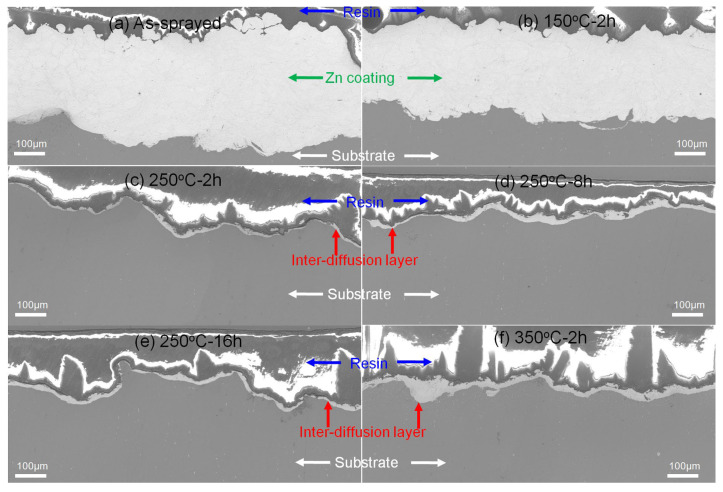
Cross-sectional microstructures of fractured surfaces of Zn coatings after different heating conditions.

The fracture surfaces of the tensile samples were investigated as shown in Figure 6. In the as-sprayed Zn coating and the Zn coating heated at 150 °C for 2 h, EDS results only show Zn and O atoms on the fracture surfaces as shown in Figure 6a,b. In addition to the locally smooth facets (tabbed as red hollow arrows), these were some typical dimples with different sizes (tabbed as red arrows) in Figure 6a,b. Their results indicate the metallurgical bonding between the deposited particles and the local ductile fractures [13,36]. It can be considered that the fractures occurred along with the inter-particle interfaces under the coating subsurface rather than the coating/substrate interfaces. In Figure 6c–f, a lot of smooth fracture regions with small facets (tabbed as yellow arrows) were the main features in addition to slight dimples (tabbed as green arrows). EDS results show that the fracture surfaces were mainly composed of Mg and Zn atoms in addition to Al and O atoms. It indicates the fractures occurred within the inter-diffusion layers in Figure 6c–f. Figure 7 shows the fracture regions of Zn coatings after tensile testing. The as-sprayed Zn coating and the Zn coating at150 °C for 2 h presented the residual Zn coatings on the substrate as shown in Figure 7a,b. However, only thin inter-diffusion layers remained on the surface of the Mg alloy substrate for Zn coatings at250 °C-2 h, 250 °C-8h, 250 °C-16h, and 350 °C-2 h as shown in Figure 7c–f. This result further confirms that crack propagations occurred along the inter-diffusion layer for the Zn coatings at a heat temperature higher than 250 °C. As shown in Figure 3, the inter-diffusion layer becomes thicker with the heat temperature and time. It is reported that the Mg-Zn intermetallic compounds present high microhardness but low fracture strength [24]. Therefore, it can be considered that the inter-diffusion layer between Zn coatings and Mg alloy substrate was the main factor in the tensile strength of Zn coatings after heat treatment.

### 3.5. Polarization Curves

**Figure 8:**
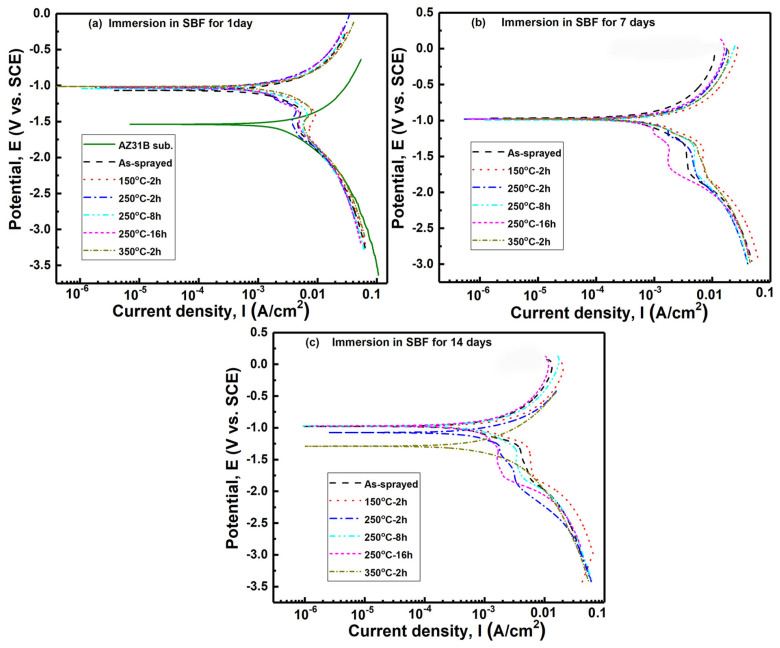
Polarization curves of Zn coatings after different immersion time.

Figure 8 shows polarization curves for Mg alloy substrates and Zn coatings before and after heating after immersion in SBF for a different time. Due to the serious corrosion, the polarization curves of the Mg alloy substrate were not presented after immersion for 7 and 14 days. Polarization curves were fitted by Tafel extrapolation method and the corrosion rate was calculated according to the literature [37]. Table 1 shows the fitting results of the corrosion current density, the corrosion potential, and the corrosion rate for different specimens. After immersion in Hanks’ SBF for 1 day, both the as-sprayed and post-heated Zn coatings improved the anti-corrosion property of the Mg alloy substrate with lower corrosion current, lower corrosion rate, and higher corrosion potential. Both the heat temperature and heat time presented a slight improvement in the anti-corrosion properties of the Zn coatings. As shown in Figure 1 and Figure 2, the phase compositions and microstructures of Zn coatings were comparable before and after heating. This result indicates that the cold-sprayed Zn coatings can protect the Mg alloy substrate from corrosion. Increasing the immersion time, the Zn coatings presented different corrosion properties. After immersion for 7 days, the corrosion potential was comparable to all the Zn coatings, while the corrosion current and corrosion rate of all the Zn coatings were decreased by comparing with immersion for 1 day. Some Zn coatings after heating presented a slightly higher corrosion current and corrosion rate than the as-sprayed Zn coating. However, after immersion for 14 days, Zn coatings before and after heating show comparable anti-corrosion properties with lower corrosion current and lower corrosion rate. By extending the immersion time, Zn-based corrosion products on the surfaces of coatings can also hinder the penetration of the corrosion medium [6,7,8]. There is a difference from the reported result of significant improvements in the corrosion resistance of the Zn coating after post-heating [11]. This difference can be attributed to the dense microstructures in the as-sprayed Zn coating with severe particle flattening induced by cold spray assisted by in-situ micro-forging [28,29,38]. However, the post-heat treatment presented a limit of improvement in densifications of the Zn coatings as shown in Figure 2, except for 350 °C for 2 h. The present Zn coatings before and after heating effectively improve the anti-corrosion property of the Mg alloy substrate, which is consistent with our previous results [14,15]. It can be considered that post-heat treatment presented a slight improvement in the anti-corrosion properties of Zn coating, and the anti-corrosion properties of Zn coatings could be improved with increasing immersion time.

### 3.6. Corrosion Behavior

**Figure 9:**
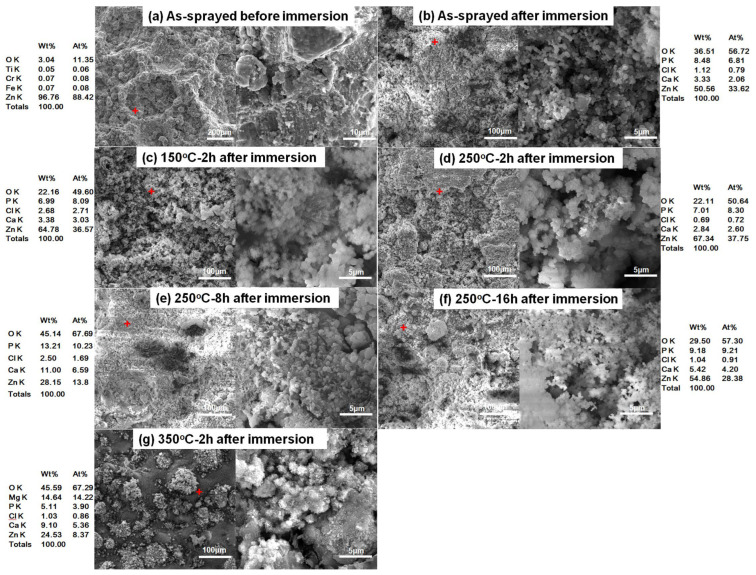
Surface morphologies of different Zn coatings after immersion for 14 days. Red plus sign: location for EDS.

**Figure 10:**
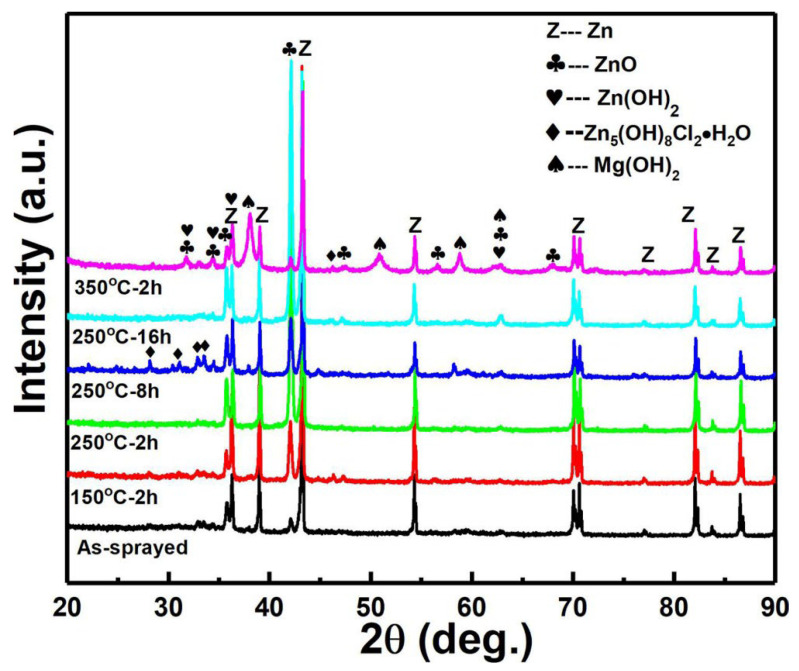
XRD patterns of different Zn coatings after immersion for 14 days.

Figure 9 shows the surface morphologies of different Zn coatings before and after immersion in SBF for 14 days. For the as-sprayed Zn coating, a rough surface with near-spherical Zn particles and large carters induced by shot impacts were observed in Figure 9a. Some melting features were found in the magnified image, which is similar to the reported result [13]. After immersion for 14 days, the surface features on as-sprayed Zn coatings disappeared, as shown in Figure 9b–g. All the Zn coatings exhibited comparable surface morphologies with tiny spherical and agglomerating granular corrosion products. EDS results show that the corrosion products were mainly composed of Zn, O, Cl, P, and Ca. There was a lot of Mg on the Zn coating heated at 350 °C for 2 h as shown in Figure 9g. The XRD patterns show that in addition to Zn and ZnO, Zn(OH)_2_ and Zn_5_(OH)_8_Cl_2_·H_2_O existed on all the surfaces of Zn coatings, as shown in Figure 10. The intensity of the ZnO phase was increased in all the coatings after immersion. The corrosion products of ZnO, Zn(OH)_2,_ and Zn_5_(OH)_8_Cl_2_·H_2_O could be formed by the Zn dissolution and chemical reaction between Zn^2+^, Cl^-^, and OH^-^ [39,40]. In addition, the Mg(OH)_2_that existed on the Zn coating was heated at 350 °C for 2 h, which could be formed by the corrosion of the Mg alloy substrate.

**Figure 11:**
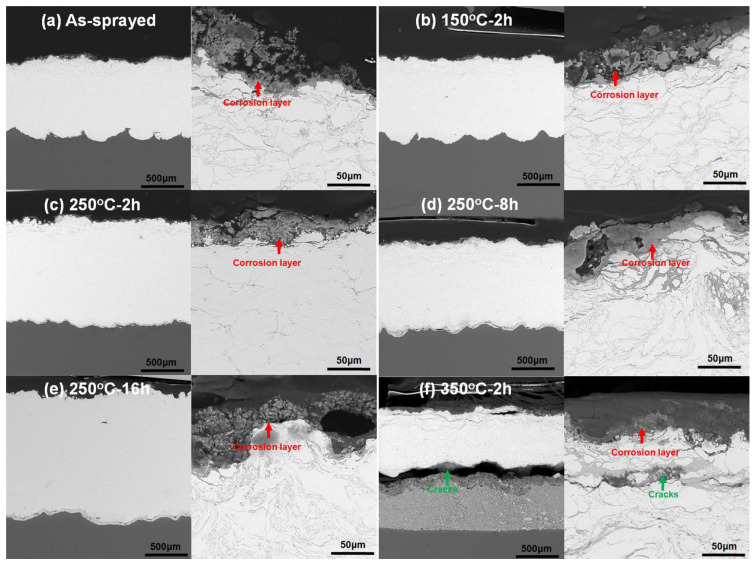
Cross-sectional microstructures of different Zn coatings after immersion for 14 days.

Figure 11 shows cross-sectional microstructures of all the Zn coatings after immersion for 14 days. It can be found that thin and gray layers of porous corrosion products covered all the Zn coatings. In addition to the corrosion layers, there was no sign of the penetration of the corrosion medium into the substrates in Figure 11a–e. It indicates that the as-sprayed Zn coatings and Zn coatings heated at less than 350 °C could protect the Mg alloy substrates from corrosion. However, in addition to small cracks under the subsurface, a big crack existed at the coating/inter-diffusion layer interface in the Zn coating when heated at 350 °C for 2 h in Figure 11f. The corrosion of the Mg alloy substrate in Figure 11f could be attributed to the pores and cracks in the Zn coating, as shown in Figure 2f. Although there was no visual sign of corrosion medium penetrating from the coating to the substrate in Figure 11f, it is reasonably inferred that pores and cracks in the coating (in Figure 2f) acted as channels for the penetration of the corrosion medium. Due to the difference in the corrosion potential between the Mg-Zn intermetallic compounds and the Zn coating [21], there was a big crack between the inter-diffusion layer and the Zn coating. Therefore, it can be considered that except for the Zn coating heated at 350 °C for 2 h, Zn coatings after heat treatment can hinder the corrosion medium by penetrating into the Mg alloy.

## 4. Conclusions

The influence of post-process heat treatment on microstructure, mechanical, and anti-corrosion properties of cold-sprayed Zn coatings was systematically investigated. The Zn coatings presented similar phase compositions of Zn and ZnO before and after heat treatment, but the full width at half maxima of the Zn phase was decreased. Except for the heat condition of 350 °C for 2 h, Zn coatings presented comparable microstructures after heat treatments. Inter-diffusion layers with different thicknesses were formed at the coating/substrate interfaces. The lowest thickness was 2.2 ± 0.4 μm at 150 °C for 2 h, and the largest thickness was 704.1 ± 32.4 μm at 350 °C for 2 h. The Zn coatings, the substrate, and the inter-diffusion layers exhibited different evolutions of microhardness with the heat temperature and time. The microhardness of Zn coatings was significantly decreased from 70.8 ± 5.6 to 43.9 ± 12.5 HV_0.025,_ with the heat temperature from the ambient temperature to 350 °C, and was slightly decreased from 65.1 ± 12.7 to 58.6 ± 4.7 HV_0.025,_ with the heat time from 250 °C for 2 h to 250 °C for 16 h. The inter-diffusion layer presented the lowest microhardness of 125.2 ± 24.6 HV_0.025_ at 150 °C for 2 h and the highest microhardness of 323.7 ± 104.1 HV_0.025_ at 350 °C for 2 h. The tensile strength of Zn coatings after heating was relative to the inter-diffusion layers and was decreased with the heat temperature and time. The highest tensile strength was 40.9 ± 3.9 MPa at 150 °C for 2 h, and the lowest thickness was 6.6 ± 1.6 MPa at 350 °C for 2 h. Zn coatings before and after heat treatment showed lower corrosion currents and lower corrosion rates than the Mg alloy substrate, but the effect of heat treatment was limited. The lowest corrosion current and corrosion rate were 0.62 ± 0.23 mA/cm^2^ and 7.3 ± 3.5 mm/a for the Zn coating heated at 150 °C for 2 h after immersion for 14 days, respectively. Except for the Zn coating heated at 350 °C for 2 h presenting cracks at the coating/substrate interface, Zn coatings before and after heat treatment effectively hindered the simulated body fluid penetrating into the substrate with porous corrosion products on the Zn coating surfaces after immersion for 14 days.

## Figures and Tables

**Table 1:** Fitting results from polarization curves in Figure 8.

Samples	Immersion Time	β_a_ (mV)	β_c_ (mV)	I_corr_ (mA/cm^2^)	E_corr_ (V)	Corrosion Rate (mm/year)
Substrate	1 day	690.6 ± 59.9	417.6 ± 144.3	3.12 ± 0.75	−1.54 ± 0.01	29.9 ± 0.9
As-sprayed	1 day	503.4 ± 122.2	331.5 ± 53.1	1.31 ± 0.71	−1.05 ± 0.04	18.9 ± 7.7
	7 days	507.3 ± 331.4	312.2 ± 60.1	0.74 ± 0.25	−1.06 ± 0.12	8.7 ± 3.7
	14 days	540.6 ± 197.1	369.7 ± 66.5	1.07 ± 0.38	−1.11 ± 0.18	9.9 ± 4.6
150 °C-2 h	1 day	330.1 ± 204.3	254.6 ± 128.2	1.57 ± 0.72	−1.04 ± 0.04	18.5 ± 7.6
	7 days	1123.1 ± 363.9	243.2 ± 115.4	1.17 ± 0.88	−1.03 ± 0.07	12.5 ± 7.5
	14 days	522.5 ± 119.8	433.2 ± 146.5	0.62 ± 0.23	−1.02 ± 0.32	7.3 ± 3.5
250 °C-2 h	1 day	619.6 ± 236.7	374.6 ± 116.9	1.92 ± 0.49	−1.04 ± 0.05	19.9 ± 5.7
	7 days	725.9 ± 106.5	283.2 ± 73.9	1.14 ± 0.26	−0.99 ± 0.16	13.5 ± 3.1
	14 days	590.1 ± 193.4	386.4 ± 113.1	0.82 ± 0.36	−1.12 ± 0.17	9.7 ± 4.2
250 °C-8h	1 day	505.5 ± 232.9	309.1 ± 17.7	1.79 ± 0.29	−1.02 ± 0.02	21.1 ± 3.5
	7 days	516.5 ± 233.1	307.9 ± 22.5	1.58 ± 0.61	−0.99 ± 0.01	18.6 ± 7.2
	14 days	482.5 ± 61.1	433.7 ± 201.7	0.73 ± 0.25	−1.14 ± 0.24	8.6 ± 2.9
250 °C-16h	1 day	455.9 ± 23.9	468.8 ± 285.8	1.98 ± 1.06	−1.06 ± 0.05	21.3 ± 2.5
	7 days	726.1 ± 54.6	257.5 ± 123.5	0.78 ± 0.15	−0.98 ± 0.02	9.2 ± 2.6
	14 days	567.4 ± 69.3	277.1 ± 59.8	0.67 ± 0.16	−0.97 ± 0.04	7.9 ± 1.9
350 °C-2 h	1 day	383.5 ± 133.1	334.1 ± 157.7	1.47 ± 0.61	−1.17 ± 0.26	17.3 ± 7.1
	7 days	592.2 ± 157.5	282.2 ± 67.9	1.14 ± 0.24	−0.98 ± 0.02	13.4 ± 4.7
	14 days	352.7 ± 59.2	357.6 ± 78.6	0.67 ± 0.22	−1.29 ± 0.15	7.9 ± 2.3

## Data Availability

Not applicable.
